# Differential Impacts of HHV-6A versus HHV-6B Infection in Differentiated Human Neural Stem Cells

**DOI:** 10.3389/fimmu.2022.847106

**Published:** 2022-07-13

**Authors:** Elham Bahramian, Mercede Furr, Jerry T. Wu, Ruben Michael Ceballos

**Affiliations:** ^1^ Department of Biological Sciences, University of Arkansas, Fayetteville, AR, United States; ^2^ Cell and Molecular Biology Program, University of Arkansas, Fayetteville, AR, United States; ^3^ Department of Biology, Johns Hopkins University, Baltimore, MD, United States; ^4^ Ecology, Evolution, and Organismal Biology Group, University of Arkansas, Fayetteville, AR, United States; ^5^ Arkansas Center for Space and Planetary Sciences, University of Arkansas, Fayetteville, AR, United States

**Keywords:** human herpesvirus 6, cell tropism, immunological response, neural stem cells, epilepsy, roseolovirus

## Abstract

Within the family *Herpesviridae*, sub-family *β-herpesvirinae*, and genus *Roseolovirus*, there are only three human herpesviruses that have been described: HHV-6A, HHV-6B, and HHV-7. Initially, HHV-6A and HHV-6B were considered as two variants of the same virus (i.e., HHV6). Despite high overall genetic sequence identity (~90%), HHV-6A and HHV-6B are now recognized as two distinct viruses. Sequence divergence (e.g., >30%) in key coding regions and significant differences in physiological and biochemical profiles (e.g., use of different receptors for viral entry) underscore the conclusion that HHV-6A and HHV-6B are distinct viruses of the *β-herpesvirinae*. Despite these viruses being implicated as causative agents in several nervous system disorders (e.g., multiple sclerosis, epilepsy, and chronic fatigue syndrome), the mechanisms of action and relative contributions of each virus to neurological dysfunction are unclear. Unresolved questions regarding differences in cell tropism, receptor use and binding affinity (i.e., CD46 versus CD134), host neuro-immunological responses, and relative virulence between HHV-6A versus HHV-6B prevent a complete characterization. Although it has been shown that both HHV-6A and HHV-6B can infect glia (and, recently, cerebellar Purkinje cells), cell tropism of HHV-6A versus HHV-6B for different nerve cell types remains vague. In this study, we show that both viruses can infect different nerve cell types (i.e., glia versus neurons) and different neurotransmitter phenotypes derived from differentiated human neural stem cells. As demonstrated by immunofluorescence, HHV-6A and HHV-6B productively infect VGluT1-containing cells (i.e., glutamatergic neurons) and dopamine-containing cells (i.e., dopaminergic neurons). However, neither virus appears to infect GAD67-containing cells (i.e., GABAergic neurons). As determined by qPCR, expression of immunological factors (e.g., cytokines) in cells infected with HHV-6A versus HHV6-B also differs. These data along with morphometric and image analyses of infected differentiated neural stem cell cultures indicate that while HHV-6B may have greater opportunity for transmission, HHV-6A induces more severe cytopathic effects (e.g., syncytia) at the same post-infection end points. Cumulatively, results suggest that HHV-6A is more virulent than HHV-6B in susceptible cells, while neither virus productively infects GABAergic cells. Consistency between these *in vitro* data and *in vivo* experiments would provide new insights into potential mechanisms for HHV6-induced epileptogenesis.

## Introduction

Human herpesvirus 6 (HHV-6) variants fall into two sub-groups of viruses, which are now distinguished by the International Committee on Taxonomy of Viruses as distinct virus species, designated as HHV-6A and HHV-6B, in the genus *Roseolovirus* (1). Along with human herpesvirus 7 (HHV-7), HHV-6A and HHV-6B are the only characterized human viruses included within the genus *Roseolovirus* (subfamily *β-herpesvirinae*, family *Herpesviridae*). Despite exhibiting approximately 90% overall genome sequence identity (2), key regions of the HHV-6A and HHV-6B genomes (i.e., immediate-early genes) exhibit only 70% (or less) sequence identity (3). Beyond notable gene sequence divergence, key coding regions yield homologous proteins with distinguishable amino acid profiles even when the genes exhibit high (e.g., 95%) sequence homology (4, 5). For example, HHV-6A and HHV-6B envelope glycoproteins (i.e., gH and gB), which are critical for virus-cell surface interactions, exhibit overall high sequence identity between homologous genes but feature distinct amino acid profiles for each of the two species (5). This may account for noted variations in cell tropism (and etiology) between the two viruses (6, 7). Until 2013, an inhibitory complement receptor, CD46 (cluster of differentiation 46) was considered to be the primary target for HHV-6A attachment to susceptible cell types (8, 9). However, it has been suggested that tumor necrosis factor receptor superfamily member 4 (TNFRSF4), also known as CD134, serves as the primary receptor for HHV-6B entry into susceptible cell types (10). Indeed, there may be other yet to be identified cell surface receptors to which HHV-6 virions can bind with asymmetric binding affinity between such receptors and HHV-6A versus HHV-6B envelope glycoproteins. This area of research is ongoing.

Among the tissues and organs that are known to harbor HHV-6A and HHV-6B, it is known that both viruses can infect the central nervous system. (11, 12). Although reports suggest that HHV-6A may be more neurotropic than HHV-6B (13), this is primarily based on a prevalence of HHV-6A over HHV-6B in cerebral spinal fluid and blood of patients with rhomboencephalitis, multiple sclerosis, and other neuroinflammatory diseases (14, 15). In general, there is a lack of evidence showing the extent of HHV-6A versus HHV-6B cell tropism in the central nervous system (CNS). It is unknown whether the brain proper or nerve cells within the spinal column exhibit predisposed susceptibility to one virus over the other. In the brain, it is unknown if there is predisposition for HHV-6A versus HHV-6B infection in glia versus neurons. Once it became clear that HHV-6A and HHV-6B were two distinct viruses, it was demonstrated that both were able to infect astrocytes (16). Since then, a few studies have emerged differentiating between HHV-6A and HHV-6B infection in select nerve cell types (17, 18). However, such studies are limited (*see review* 19). The relative virulence of HHV-6A versus HHV-6B on susceptible nerve cell types and differential susceptibility of specific neuronal neurotransmitter phenotypes to these two viruses remains unclear. Characterization of infection dynamics and cell tropism of HHV-6A versus HHV-6B is essential for validating models of HHV6-based neurological dysfunction. Recently, a study was published demonstrating that HHV-6A and HHV-6B infect Purkinje cells (17). Although Purkinje cells are GABAergic, this study did not detect productive HHV-6A or HHV-6B infection in GABAergic cells. In this study, we provide data from an immunofluorescence (and qPCR) study confirming that both HHV-6A and HHV-6B infect both GFAP-positive cells (i.e., glia) and βIII tubulin-positive cells (i.e., neurons) with notable cytopathic effects. We also show that both viruses can infect different neuronal neurotransmitter phenotypes. However, neither HHV-6A nor HHV-6B appears to infect GABAergic cells. In cells which are susceptible, the severity and onset time for cytopathic effects differs between HHV-6A versus HHV-6B infections. HHV-6A and HHV-6B also appear to differentially impact expression of cytokines and growth factors associated with viral infection.

## Materials and Methods

### Cell Culture

Culture vessels (i.e., T75 flasks and 8-well microscope chamber slides; Thermofisher Scientific) were coated with CELLStart™ surface substrate (Gibco, Life Technologies Corporation) per supplier protocol. In serum-free medium (SFM) [Knockout™ DMEM/F-12, 20 ng/mL FGF-2/EGF, 2mM GlutaMAX™-I, and 2% v/v StemPro™ Neural supplement], NIH-approved H9-derived human embryonic stem cells (hESCs) were plated and expanded as a monolayer in CELLStart™ coated vessels. The cultures were maintained at 37°C in a humidified incubator (with 5% CO_2_). Cells were passaged every 7 days using an accutase cell detachment solution (Sigma-Aldrich) and re-plating at a surface density of 5 x 10^4^ cells/cm^2^. Using an automated cell counter (Bio-Rad) and Trypan Blue (Thermofisher Scientific), viable cell counts were determined for re-plating cells. Upon re-plating, different media options were used to induce differentiation along desired paths. For example, to facilitate differentiation toward neuron-dense mixed cultures, a seeding density of 2.5 x 10^4^ cells/cm^2^ was used with a minimal media (MM) [Knockout™ DMEM/F-12, 2mM GlutaMAX™-I, and 2% StemPro™ Neural Supplement]. Cells were maintained in differentiating conditions for 15-18 days with fresh media change-out every 3-4 days.

### Virus Preparation

Frozen stocks (-195°C) of HHV-6A strain GS-infected HSB2 cells and HHV-6B strain Z29-infected MOLT-3 cells (courtesy NIH) were thawed and used to infect uninfected HSB2 and MOLT-3 cells. Specifically, 10^6^ HHV6-infected cells were mixed with uninfected cells at a ratio of 1:10 in a T150 flask (Thermofisher Scientific) and incubated at 37°C for 2 hr. (5 mL of media). After 2 hr of incubation fresh media as added (5 mL) and the cells were incubated again at 37°C. Using a light microscope, the culture was checked periodically. When cytopathic effects (CPEs) were noted in more than 80% of cells, the cell suspension was harvested. Aliquots were stored in liquid nitrogen (-195°C) for use in infection assays. Alternatively, cell-free virus suspension was prepared by sonicating cell suspension on ice and centrifuging the lysate at 3500 rpm for 1 hr to pellet cell debris while maintaining virus particles in suspension. The supernatant was extracted and filtered through a 0.45μm filter to remove any remaining cell debris. The virus-containing filtrate was centrifuged at 25,000 rpm at 4°C for 3 hr to pellet the virus. Supernatant was removed and the virus pellet was resuspended in cold media and stored at -80°C for use in cell-free virus infection assays or for transmission electron microscopy. Virus titers were determined using qPCR.

### Transmission Electron Microscopy for Cell-Free Virus

Transmission electron microscopy (TEM) was used for three purposes. First, TEM was used to verify the presence of intact, fully assembled virions from HSB2 and MOLT-3 host cells used for virus storage and propagation. Second, TEM was used to demonstrate the presence of HHV6 virus particles in differentiated human neural stem cells (dHNSC). Third, TEM was used to validate the presence of virus for qPCR-based titers from storage cells or dHNSC. For cell-free virus samples, the aforementioned preparation *via* sonication (or, alternatively, freeze-thaw cycles) was followed by concentration of virus suspensions (i.e., filtered lysates) using 30kDA MWCO spin concentrator (Sigma-Millipore). From the retentate, ~5μL of concentrated viral suspension was spotted onto a formvar-coated copper grid and incubated for 10 min in a humidity chamber. The sample was then rinsed and negatively stained with a 2% uranyl acetate solution for 30 sec before excess solution was wicked from the grid and allowed to air dry for 1 hr. Samples placed on grids, and after 2 to 10 minutes, 2% uranyl acetate was added to the grid. Grids were imaged with a Hitachi H-7100 TEM at 75 kV. Images were captured at 60,000– 200,000X magnification.

### Transmission Electron Microscopy for Infected Cells

TEM was also used to image virus particles associated with host cells. These included both virus storage cells (HSB2 and MOLT-3 cells) and dHNSC infected with either virus. For virus-infected storage cells in or for virus-infected monolayers of dHNSC, cells were fixed with a 4% solution of paraformaldehyde (PFA). The samples were place on grids and then gently rinsed and negatively stained with a 2% solution of uranyl acetate. Samples were incubated for 2 hr at 4°C before rinsing with distilled water and wicking off excess solution from the grid and allowed to air dry for 1 hr. After air drying for 2 hr., samples were imaged with a Hitachi H-7100 TEM at 75 kV. Images were captured at 60,000– 200,000X magnification. Regions of the grid that showed virions blebbing from membrane or virus particles within the intracellular space were targeted for imaging. Detachment of HNSC from the surface substrate was required, to capture co-localization of virus particles within intact cells.

### Light Microscopy

The infected cultures were viewed daily *via* light microscopy to monitor morphological changes. (In each experiment, uninfected cultures served as control). Light microscopy images were taken from dHNSC at post-differentiation day 7 (PDD7) and PDD14 at 2 hours post-infection (HPI) and 24 HPI using an upright microscope (Primovert, Zeiss) with a color camera (AxioCam 105, Zeiss). Dozens of cells were captured in each image from randomly chosen fields of view for each culture.

### mRNA Profiling

RNA extraction kit was used to analyze the mRNA profile of dHNSC (Qiagen, Hilden, Germany). Total cellular RNA was collected from uninfected cells at PDD7 and cells infected with HHV-6A and HHV-6B at PDD7 as well as PDD14. A DNase treatment using a kit was performed on samples to eliminate DNA contamination (Ambion™ DNase I). cDNA was synthesized using (iScript Advanced cDNA Synthesis Kit for RT-qPCR, BioRAD, US). H9 cell genomic DNA was used (Qiagen DNA extraction kit), and PCR was performed using a particular primer for each cytokine and growth factor (**Table S1**). Primer for each gene was designed through NCBI and ordered from the IDT website (http://www.idtdna.com). The PCR product concentration was determined using a nanodrop and PCR product was used at known concentrations to generate a standard curve for calculating each individual gene expression levels. Serial dilutions for each target gene were performed in triplicate. The standard serves to calculate transcript levels of the same gene in unknown samples using a linear series (log scale). Because both standard and unknown samples were used in the same run, experimental errors are minimized. qPCR primer sequence sets (both forward and reverse) for each target (e.g., IL-1β, IL-6, IL-10, TLR9, TNFα) are provided (*see* **Supplementary Table 1**). The following formula was used to determine transcript copy number:


$$\text{number of copies}\left(\text{molecules}\right)=\frac{X\text{ ng}\left(6.0221\times 10^{23}\text{ molecules}/\text{mole}\right)}{N\left(660\text{ g}/\text{mole}\right)\;(1\times 10^{9}\text{ng}/\text{g)}}$$

such that, *X* represents the amount of amplicon in nanograms, *N* is the length of the dsDNA amplicon, and 660 g/mole is the average mass of 1 bp dsDNA.

### Immunofluorescence and Fluorescence Microscopy

Immunofluorescence was conducted *via* a co-labeling approach (two antibody systems per trial) along with the nuclear dye 4,6-diamidino2-phenylindole dihydrochloride (DAPI). The following fluorescence antibody systems (primary antibody/secondary antibody) were used in select pairs: mouse anti-gB (HHV6 envelope glycoprotein gB)/donkey anti-mouse IgG-Alexa Fluor 488; chicken anti-GFAP (Glial Fibrillary Acid Protein)/donkey anti-chicken IgG-Alexa Fluor 680; rabbit anti-βIII tubulin (neuron-specific microtubule protein)/donkey anti-rabbit IgG-Alexa Fluor 568; goat anti-VGluT (vesicular glutamate transporter protein)/donkey anti-goat IgG-Alexa Fluor 555; chicken anti-GAD67 (glutamate decarboxylase 67)/donkey anti-chicken IgG-Alexa Fluor 680; and, rabbit anti-DA (dopamine)/donkey anti-rabbit IgG-Alexa Fluor 568. The anti-gB antibody (courtesy NIH AIDS reagent program) and fluorescent secondary indicate the presence of HHV6 virus and when co-localized with other immunofluorescence markers demonstrate infection in select cell types (i.e., neurons versus glia or distinct neuronal neurotransmitter phenotypes). Signal for anti-gB is usually color-coded green in alignment with emissions during image analysis. Signal for the anti-βIII tubulin fluorescent antibody system (Sigma-Aldrich) is typically color-coded red in alignment with emissions when anti-gB is co-labeled but may also be color-coded green in uninfected controls. Signal for the anti-GFAP fluorescent antibody system (Sigma-Aldrich) is typically color-coded red in alignment with emissions but may also be color-coded green in uninfected controls or when used as a co-label for distinguishing between GFAP-positive cells and βIII tubulin-positive cells in the same image. Signal for anti-VGluT (Abcam), anti-GAD67 (Abcam), and anti-DA (EMD Millipore) fluorescence is typically color-coded red to distinguish neuronal neurotransmitter phenotype from any anti-gB signal (green). All experimental trials used DAPI (blue color code) to locate nuclei of all cells in the mixed cultures regardless of cell type. For all immunofluorescence trials, dHNSC were fixed with 4% PFA in Dulbecco’s phosphate buffer solutions (DPBS), then rinsed (3X) with DPBS. Blocking solution (5% donkey serum, 0.1% triton in DPBS) was added for 30 min. at room temperature (RT) after which primary antibodies (abs) were applied. Cells were then incubated for 24 hrs. followed by a series of rinses (3X) with DPBS and then application of secondary antibodies and further incubation. Another series of rinses (3X) with DPBS was followed by application of DAPI (0.2 nM) and a 20 min incubation at RT. The plates were subjected to a final rinse in DPBS and staged within the confocal fluorescent microscope (Leica). Image were taken using 10X magnification to view the distribution of fluorescent signals across the plates. In infected cultures, immunofluorescence was performed at multiple time-points during the differentiation phase (PDD7 and PDD14 preferred) and at several time-points after introduction of virus (e.g., 2HPI and 24HPI at MOI = 1 unless otherwise noted). [*Note:* MOI is used here to describe the ratio of viral genomes to cells at the initiation of infection]. Images for each fluorescent signal were captured and then images were overlayed using image analysis software to generate composite images.

### Quantitative Polymerase Chain Reaction

For quantitative polymerase chain reaction (qPCR)-based virus titers from both storage cell lines (i.e., HSB2 and MOLT-3 cells) and infected dHNSC, the HHV6-specific U22 gene was targeted. Using the primers (20): 397F (5′-TCG AAA TAA GCA TTA ATA GGC ACA CT-3′) and 493R (5′-CGG AGT TAA GGC ATT GGT TGA-3′) – a 99bp fragment of the U22 gene was amplified from viral DNA extracted from infected cell cultures. Amplification was performed using a Rotor-Gene Q real-time fluorescence detector thermocycler (Qiagen) programmed as follows: 94°C for 6 min; followed by 40 cycles of 94°C for 30 sec, 53°C for 30 sec, 72°C for 45 sec; and, then a final holding condition of 72°C for 7 min. Verification of fragment size (and run quality) was checked *via* gel electrophoresis. DNA concentration was determined using a micro-volume spectrophotometer (Denovix). The number of viral genomes for each sample was determined by comparing results to a standard curve.

To prepare standards for qPCR analyses, the following procedure was performed for each virus (i.e., HHV-6A and HHV-6B): one copy of the U22 target sequence was cloned into a commercial vector using a TOPO PCR Cloning Kit (Invitrogen) and transformed into competent *E. coli*. Enrichment of clones was followed by plasmid purification using the QIAprep Spin Miniprep Kit (Qiagen). The resulting plasmid DNA yield was measured by absorbance spectroscopy (OD_260nm_). PCR is then employed to amplify the gene of interest (i.e., U22) from the plasmid preparation. PCR product yield is quantified using the Denovix micro-volume spectrophotometer and then a sample is diluted down to 1 ng/μL which corresponds to a fragment copy number of 9.216 x 10^9^. From this, subsequent dilutions (10^-1^ through 10^-9^) are prepared in triplicate. Next, qPCR is used to amplify fragments from these serial dilutions and the standard curve is generated. For all qPCR trials, 10μL SYBR^®^ Green (Life Technologies, Foster City, USA), 1 μL of unknown DNA sample, 50 nM of forward primer, and 50 nM of reverse primer was used in 20 μL total reaction volumes.

## Results

For all immunofluorescence, light microscopy, and RT-qPCR experiments, dHNSC in culture were considered to be viable cells at post-differentiation day 7 (PDD7) and were used in experiments through PDD14. After PDD14 cell culture stability was unreliable. By PDD7, cells showed short branching neurites and/or longer processes (see **Figures 4** and **5**) and distinct soma morphotypes: oval/round (e.g., tear-drop shapes), square-like (e.g., star shapes), and triangular. There was no apparent correlation between morphotypes and nerve cell type (i.e., glia versus neuron) at PDD7. By PDD7, cells also emit electrical discharges as detected using a multi-electrode array plate (MEA2100 system, Harvard Bioscience, Inc., Germany). During the differentiation process, culture wells (surface area = 0.5 cm^2^) are seeded with 5 x 10^4^ undifferentiated cells in suspension. Culture thinning was common as cells attach to substrate and undergo the differentiation process. Cultures are considered viable for experiments if there are at least 10,000 cells at PDD7 in the culture wells. This minimum cell density at PDD7 is required since by PDD14 viable cell count decreases by ~70% in uninfected controls (N=6, p=0.0018). Although there is substantial loss in cell density over a 7-day period in cultured dHNSC, cell death rate at either PDD7 or PDD14 over any 2 to 6 hour period is negligible in uninfected cell cultures (i.e., controls). Thus, loss in cell density greater than 10% during a 2-hour period is not likely due to baseline attrition but rather to the experimental treatment (e.g., viral infection).

For cultures infected with HHV-6A, a 50% loss in cells was observed at 2 HPI (N=6, p=0.0013) for PDD7 infections. For PDD14 infections with HHV-6A, a 45% loss in viable cells was observed at 2 HPI (N=6, p=0.0018). For cultures infected with HHV-6B, a 31% loss in cells was observed at 2 HPI (N=6, p=0.0252) for PDD7 infections. For PDD14 infection with HHV-6B, a 54% cell loss was found at 2HPI (N=6, p=0.0002). Infection was typically at multiplicity of infection (MOI) of 1.

Differentiation of these H9 cells leads to emergence of mixed cultures of glia and neurons, including cells of different neurotransmitter phenotypes. Using a standard differentiation protocol (see section 2.1), a typical culture plate consists of ~80% glia and ~20% neurons (N=6, p=0.0020). In terms of specific neuronal neurotransmitter phenotypes, it was observed that VGluT1-positive and GAD67-positive cells emerged early in the differentiation process (i.e., by PDD3-PDD5); whereas, DA-positive cell clusters were observed at later stages of differentiation (i.e., PDD7). Furthermore, VGluT1-positive and GAD67-positive cells appeared to be more homogeneously distributed across plates, while DA-positive cells appeared in clusters in select areas of the plates. For this reason, cell counts were taken from areas of higher density of DA-positive neurons. Despite the emergence of distinct neuronal neurotransmitter phenotypes at different end-points, immunofluorescence (supported by RT-qPCR) was sufficient to address fundamental question regarding the ability of HHV-6A versus HHV-6B to productively infect different nerve cell types.

### Both Glia and Neurons Are Susceptible to Infection by Either HHV-6A or HHV-6B

**Figure 1:**
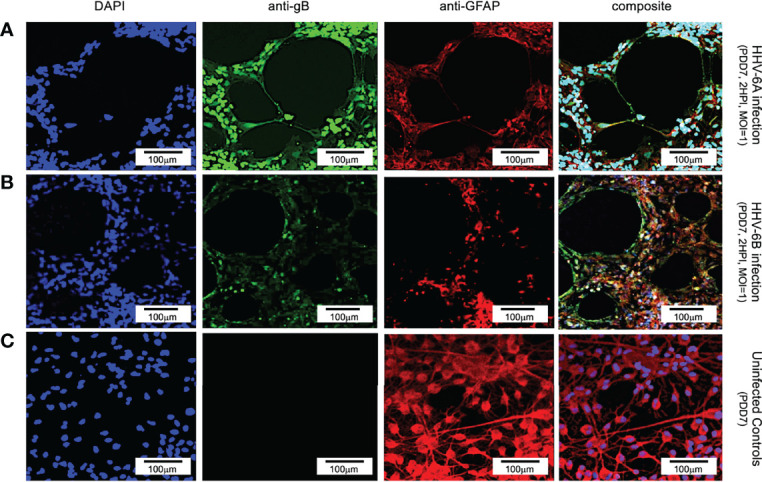
Fluorescence microscopy images of dHNSC treated with immunofluorescent antibodies and a fluoro-dye at PDD7: Differentiation to glial cells. From left to right, DAPI, anti-gB, anti-GFAP, and composites. PDD7 HNSC infected with HHV-6A (row **A**) show gB-positive signal on GFAP-positive cells (glial cells). PDD7 HNSC infected with HHV-6B (row **B**) also show gB-positive signal on GFAP-positive cells. Images for uninfected control culture (row **C**) show welldeveloped GFAP-positive cells with homogeneous distribution; many exhibit stellate morphotypes. [Scale bar = 100 micron].

Results from immunofluorescence histochemistry indicate that both HHV-6A and HHV-6B can infect cells that are positive for glial fibrillary acidic protein (GFAP) in fluorescence assays. Labeling dHNSC cultures at PDD7 with antibodies against GFAP (i.e., anti-GFAP) and HHV6 envelope glycoprotein gB (i.e., anti-gB) and staining with DAPI (4’,6-diamidino-2-phenylindole), reveals coincidence of anti-gB and anti-GFAP fluorescence signals in DAPI-stained cells (**Figure 1**). This indicates the susceptibility of glial cells to infection by HHV-6A (**Figure 1**, row A) and HHV-6B (**Figure 1**, row B). The data also show visible cell aggregation, a cytopathic effect (CPE), at 2 HPI. Distribution of cells across the substrate is notably more homogenous in the uninfected controls (**Figure 1**, row C). In these mixed cultures of dHNSC (N=12), ~58% of cells were identified as glia. Of these GFAP-positive cells, 29.2% were also gB-positive 2 HPI with HHV-6A (N=6; p=0.0201). Parallel cultures show that 42.3% of GFAP-positive cells were gB-positive in HHV-6B infected cultures (N=6; p=0.0005). Thus, results show that both HHV-6A and HHV-6B can infect glia.

**Figure 2:**
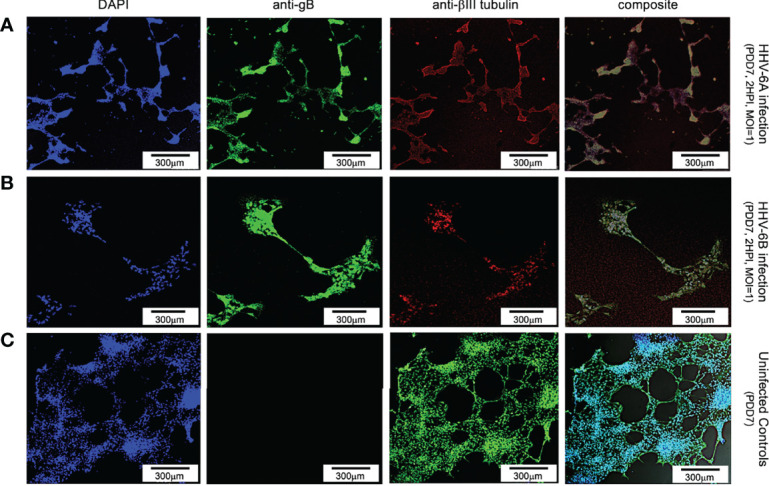
Fluorescence microscopy images of dHNSCs treated with immunofluorescent antibodies and a fluoro-dye at PDD7: Differentiation to neurons. From left to right, DAPI, anti-gB, anti-bIII tubulin, and composites. PDD7 dHNSC infected with HHV-6A (row **A**) show gB-positive signal on bIII tubulin-positive cells. PDD7 HNSC infected with HHV-6B (row **B**) also show gB-positive signal on bIII tubulinpositive cells. Images for uninfected control culture (row **C**) show developed bIII tubulin-positive cells with significant neurite-neurite and neurite-soma connectivity. [Scale bar = 300 micron].

Using an antibody for βIII tubulin (a neuron-specific protein), DAPI, and an anti-gB fluoroprobe, immunofluorescence demonstrates neurons are also susceptible to infection by both HHV-6A (**Figure 2**, row A) and HHV-6B (**Figure 2**, row B). In cells identified as neurons (i.e., βIII tubulin-positive), over 91% and 45.3% were also gB-positive at 2 HPI with HHV-6A (N=6; p=0.0081) and HHV-6B (N=6; p=0.0087), respectively. These results show that HHV-6A and HHV-6B can infect neurons. Furthermore, fluorescence images from the βIII-tubulin antibody system in uninfected cultures (**Figure 2**, row C) show elongated neurite extensions and node formation further indicating cell differentiation into viable neurons and the formation of cell-cell connectivity. At similar time points, roseolovirus infected cultures exhibit CPEs (e.g., cell aggregation and neurite disruption).

### Infection With Either HHV-6A or HHV-6B Results in Time-Dependent Cytopathic Effects

**Figure 3:**
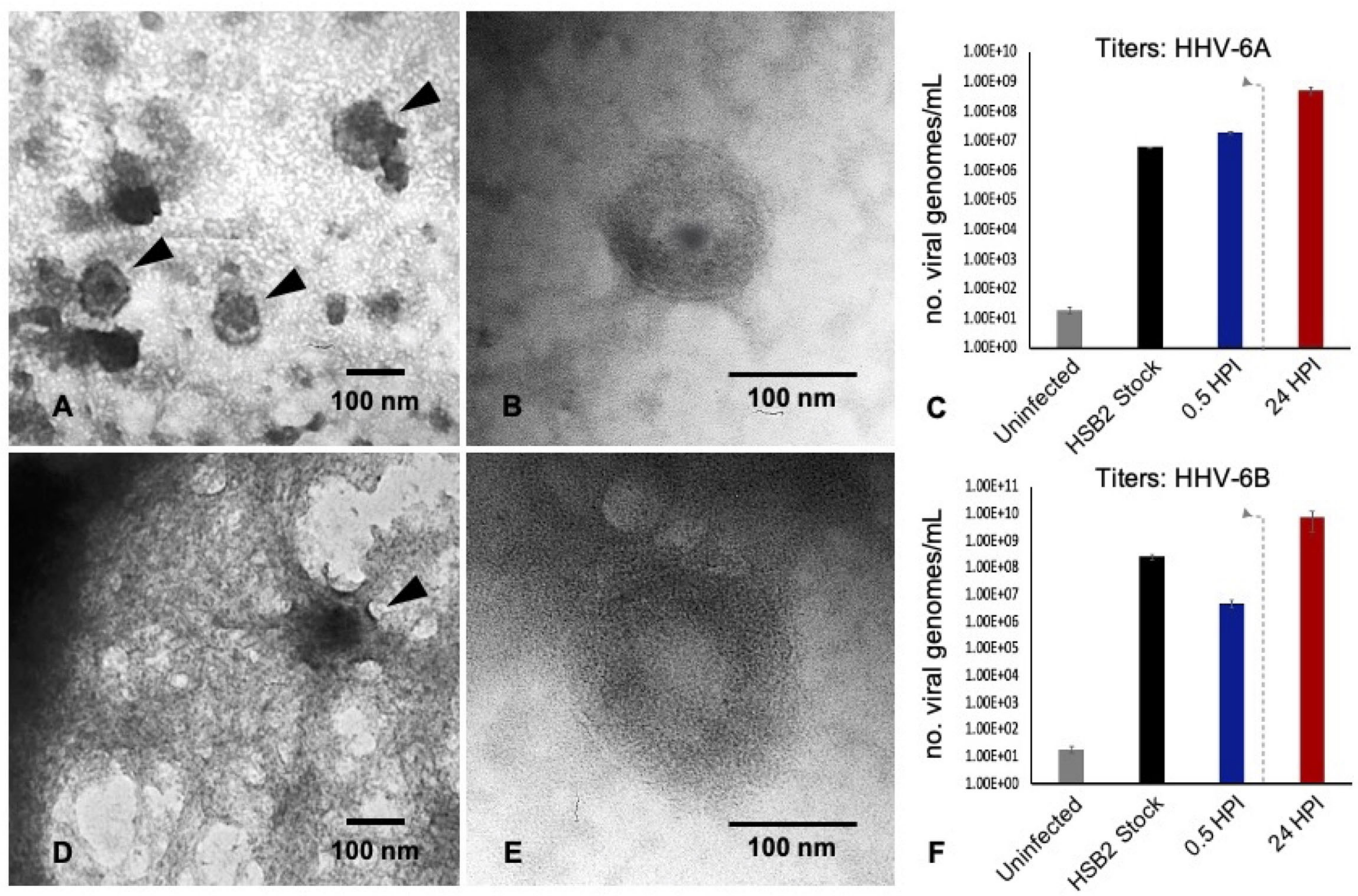
TEM and qPCR data from HHV6-infected dHNSC. TEM images illustrate: **(A)** HHV-6A virus particles within a cell; **(B)** HHV-6A virions in cell-free filtered supernatant from cell lysate; **(D)** HHV-6B virions in a cell; **(E)** HHV-6B virions in cell-free filtered supernatant. qPCR titer methods show productive virus infection for: **(C)** HHV-6A and **(F)** HHV-6B. Uninfected cells show negligible amplification of an HHV6-specific marker (i.e., U22 gene). After 30 min post-infection HHV6 is still present. After a wash at 2 HPI (dashed line) and 22 h incubation (24HPI), titers increase indicating production of progeny virions at densities greater than the number of viruses (i.e., viral genomes) present immediately after inoculation. [Scale bar = 100 nanometer].

Productive infection can be verified *via* transmission electron microscopy (TEM) and quantitative polymerase chain reaction (qPCR). TEM demonstrates the presence of fully assembled HHV-6A virus particles (**Figure 3A**) within cells that match the size and morphology of cell-free virions from virus stocks (**Figure 3B**). Likewise, HHV-6B virus particles are observed within vacuole-like spaces within dHNSCs (**Figure 3D**). These also match the size and shape of HHV-6B virions imaged from virus stocks (**Figure 3E**). To demonstrate that these are productive roseolovirus infections as opposed to a *lysis-from-without* phenomenon (21), qPCR-based virus titers (i.e., viral genome count per mL) were determined for HHV-6A (**Figure 3C**) and HHV-6B (**Figure 3F**). Within 2 HPI, the number of detectable viral genomes exceeds the virus density of the inoculum of HHV-6A and HHV-6B used to infect dHNSC cultures, thereby demonstrating productive infection (i.e., the production of progeny virus).

Together, data from TEM, immunofluorescence (using an anti-gB fluorescent antibody system), and qPCR-based quantification of virus titers at different time-points during infection provide convincing evidence that both neurons and glia are susceptible to HHV-6A and HHV-6B and result in productive viral infections. Furthermore, both TEM and immunofluorescence data indicate that infection of dHNSC by either HHV-6A or HHV-6B results in viral-induced CPEs, which manifest as disturbances to cell shape, size, viability, and distribution on culture plates. CPEs often present in two phases: cell aggregation (with notable cell death) and detachment from the culture surface.

**Figure 4:**
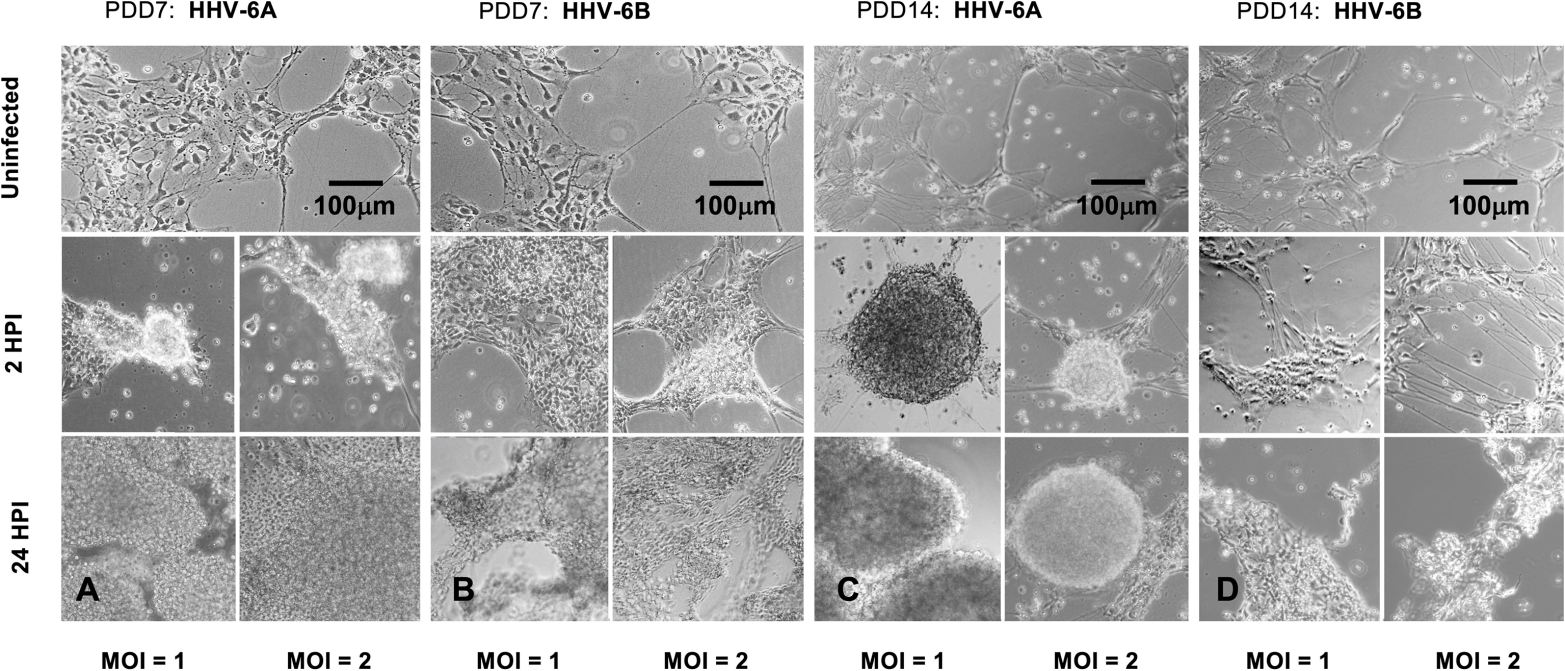
Light microscopy of HHV6-induced cytopathic effects (CPEs) on dHNSC at PDD 7 and 14. **(A)** Uninfected cultures of HNSC at PDD7 show healthy cells adhering to the plate surface (top). After two hours post-infection (2HPI) at MOIs = 1, 2 (middle, left and right, respectively) with HHV-6A, cells begin to aggregate and at 24HPI with HHV-6A at MOIs = 1, 2 (bottom, left and right) there is highdensity clumping and cell detachment. **(B)** Uninfected cultures of HNSC at PDD7 show healthy cells adhering to the plate surface (top). After 2HPI with HHV-6B at MOIs = 1, 2 (middle, left and right), cells begin to aggregate and at 24HPI with HHV-6B at MOI = 1, 2 (bottom, left and right) there is higher-density aggregation. **(C)** At PDD14, uninfected healthy cells persist (top); however, at 2HPI at MOIs = 1,2 (middle, left and right) infection with HHV-6A results in highdensity cell aggregation and at 24HPI rampant cell death and detachment is observed (bottom, left and right). **(D)** At PDD14, uninfected cells persist (top); however, HHV-6B infection at MOIs = 1,2 for 2 HPI results in lower-density cell aggregation (middle, left and right) with higher-density clumping occurring at 24HPI (bottom, left and right). [Scale bar = 100 micron].

Although there is notable aggregation of cells in both HHV-6A and HHV-6B infected cell cultures (2HPI, MOI=1 or MOI=2), HHV-6A infected cultures produce higher density clumps at earlier time points for both PDD7 and PDD14 cultures (**Figures 4A, C**, *middle row*). By 24 HPI, HHV-6A infections yield high-density detached clumps floating in culture (**Figures 4A, C**, *bottom row*). Although HHV-6B infections induce cell aggregation, cells appear more resistant to forming detached high-density spherical clumps when compared to HHV-6A infection at 2 HPI and 24 HPI (**Figures 4B, D**). In some trials, HHV-6B infected cultures featured cells that appear to retain morphological integrity at 2HPI for both PDD7 and PDD14 (**Figure 4D**). These data suggest that the severity and time-course of CPEs may differ between HHV-6A and HHV-6B infections (**Figure 4**).

**Figure 5:**
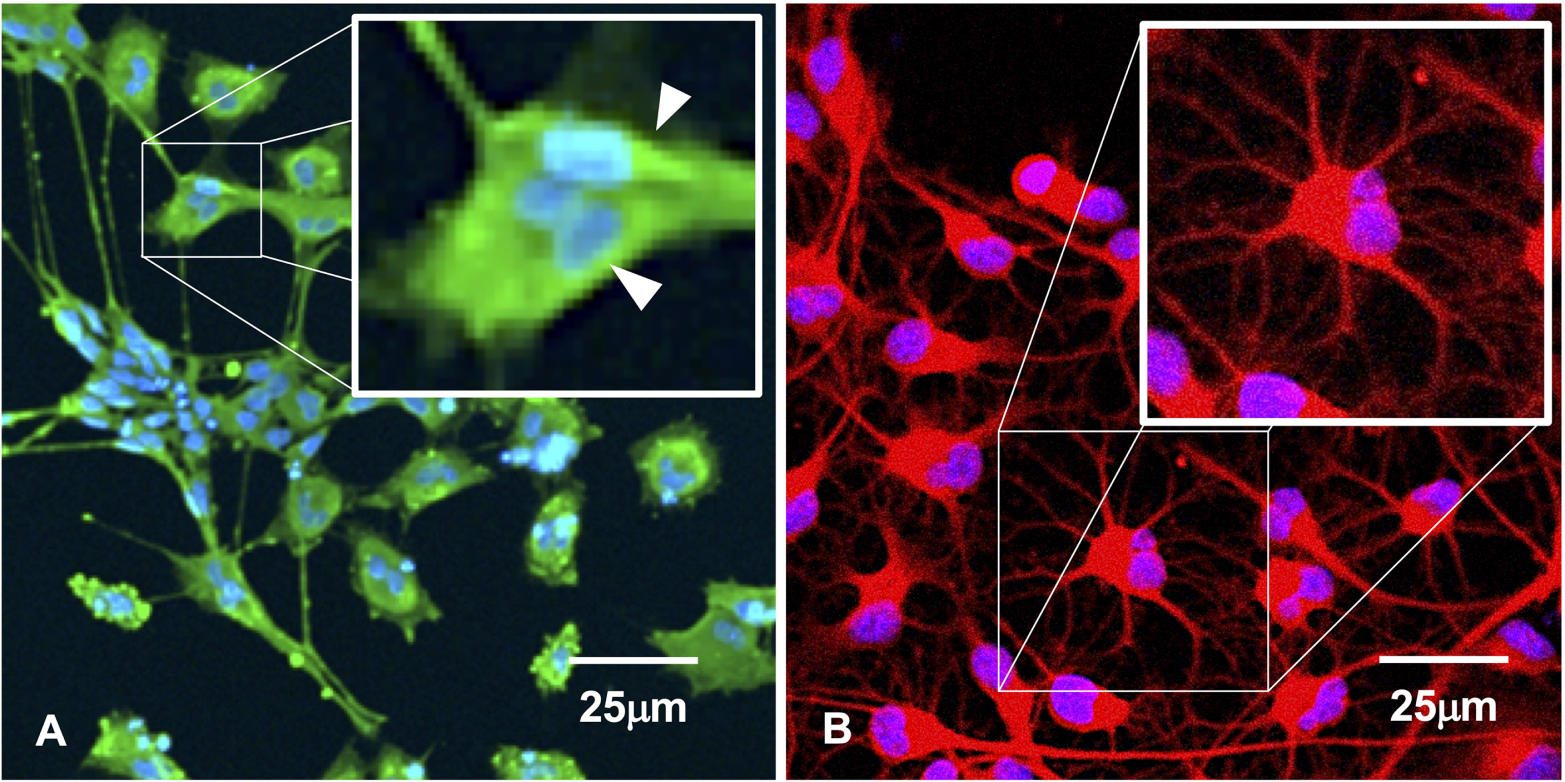
Immunofluorescence suggests syncytia. **(A)** HHV-6A infection in HNSC results in syncytia formation as indicated by cell membrane fusion and multi-nucleated cells (arrows). **(B)** Uninfected culture shows individual well-bounded dHNSC membranes and the absence of any cell aggregation that would suggest syncytia-like formations. [Scale bar = 25 micron].

To determine whether clumping of cellular biomass during the course of HHV6 infection is simply aggregation or *bona fide* viral-induced syncytia formation, fluorescence microscopy was used to detect morphological features characteristic of syncytia (**Figure 5**). Results indicate that prior to gross detachment from the culture surface (i.e., 0-2 HPI), morphological features consistent with syncytia occur, including cell fusion and formation of multi-nucleated super cells (**Figure 5A**; *arrows*). Syncytia formation was notable in HHV-6A infection. However, no discernable syncytia formation was observed during HHV-6B infection. In uninfected cultures, cells with robust arborized neurites and a single nucleus will persist for weeks (**Figure 5B**) and had slightly larger, well-defined somas.

### Both Glutamatergic and Dopaminergic Cells Are Susceptible to Either HHV-6A or HHV-6B

**Figure 6:**
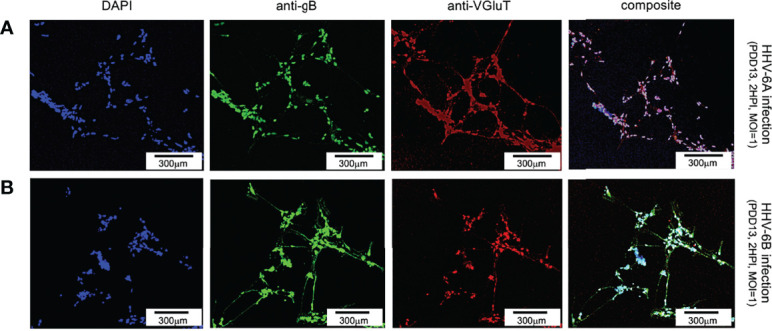
Fluorescence microscopy images of dHNSC treated with immunofluorescent antibodies and a fluoro-dye at PDD13: Glutamatergic neurons. VGluT1- positive dHNSC at PDD13 (2 HPI, MOI=1) shown (left to right) by DAPI staining, anti-gB, and anti-VGluT immunofluorescence (with composites) indicate that gB (green) colocalizes with VGluT1 (red) in DAPI stained (blue) cells for both HHV-6A (row **A**) and HHV-6B (row **B**) infected cultures, suggesting susceptibility of glutamatergic neurons to both viruses. (Results were consistent across 6 replicate trials). [Scale bar = 300 micron].

To determine if either HHV-6A or HHV-6B preferentially infects select neuronal neurotransmitter phenotypes, differentiated neurons were co-labeled with neurotransmitter-specific antibodies. For glutamatergic cells, an anti-VGluT1 fluorescent antibody system was used to target the vesicular glutamate transporter (VGluT1), a characteristic protein found in glutamatergic neurons. Simultaneous staining with DAPI and use of the anti-gB fluoroprobe shows that VGluT1-positive cells coincide with anti-gB fluorescence signals in dHNSCs infected with HHV-6A (**Figure 6**, row A) or HHV-6B (**Figure 6**, row B). This reveals that both roseoloviruses can infect of glutamatergic cells. Of cells in mixed cultures identified as glutamatergic (i.e., VGluT1-positive), 96% (N=6, p=0.0004) and 73% (N=6, p=0.0005) were gB-positive in HHV-6A and HHV-6B infections, respectively.

**Figure 7:**
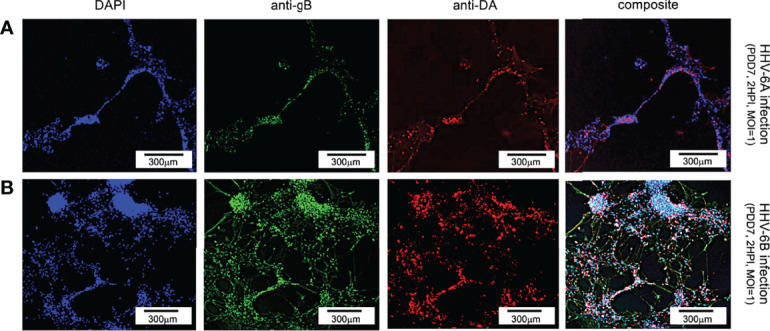
Fluorescence microscopy images of dHNSC treated with immunofluorescent antibodies and fluoro-dye (DAPI) at PDD7: Dopaminergic neurons. DA-positive dHNSC at PDD7 (2HPI, MOI = 1) shown (left to right) by DAPI staining and antigB and anti-DA immunofluorescence (with composites) indicate that gB (green) colocalizes with dopamine (red) in DAPI stained (blue) cells for both HHV-6A (row **A**) and HHV-6B (row **B**) infected cultures, suggesting susceptibility of dopaminergic neurons to both viruses (Results were consistent across 6 replicate trials). [Scale bar = 300 micron].

For dopaminergic cells, an anti-dopamine (anti-DA) fluorescent antibody system was used to directly target dopamine. Co-staining with DAPI and co-labeling with anti-gB and anti-DA, fluorescence microscopy shows a co-localization of DAPI, anti-gB, and anti-DA signals (**Figure 7**). This suggests that dopaminergic neurons are susceptible to infection by HHV-6A (**Figure 7**, row A) and HHV-6B (**Figure 7**, row B). Of all cells in mixed cultures labeled dopaminergic (i.e., DA-positive), 89% (N=6, p=0.0470) and 77% (N=6, p=0.0000) were gB-positive in HHV-6A and HHV-6B infections, respectively. This demonstrates that both viruses can infect of dopaminergic neurons. Roseolavirus appears to be widely distributed throughout infected cells. In both VGluT1-positive and DA-positive cells, anti-gB fluorescence was seen within neurites as well as cell soma.

### Neither HHV-6A nor HHV-6B Appears to Infect GAD67-Positive Cells (GABAergic neurons)

**Figure 8:**
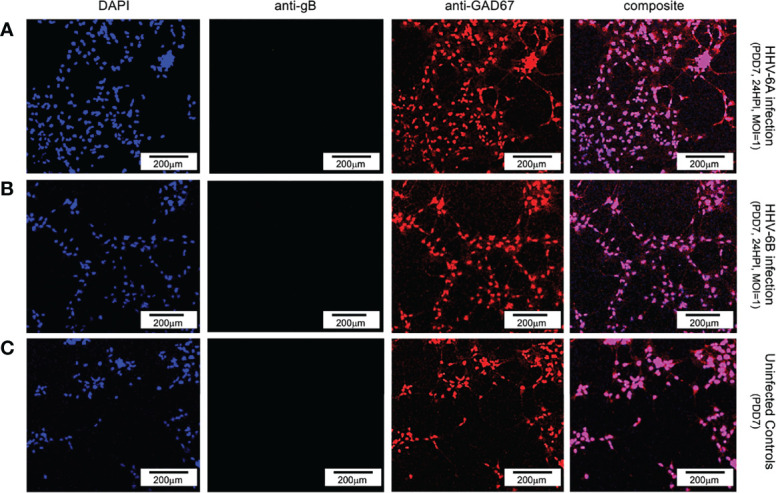
Fluorescence microscopy images of dHNSC treated with immunofluorescent antibodies and a fluoro-dye (DAPI) at PDD7: GABAergic neurons. GAD67- positive dHNSCs at PDD7 (2HPI, MOI=1) shown (left to right) by DAPI dye and anti-GAD67 immunofluorescence (with composites) indicate that gB (green) immunofluorescence does not colocalize with GAD67 (red) in DAPI stained (blue) cells for both HHV-6A (row **A**) and HHV-6B (row **B**) infected cultures, suggesting GABAergic neurons are not susceptible to either virus. No gB signal (green) is detected in uninfected controls (row **C**). (Results were consistent across 6 replicates). [Scale bar = 200 micron].

**Figure 9:**
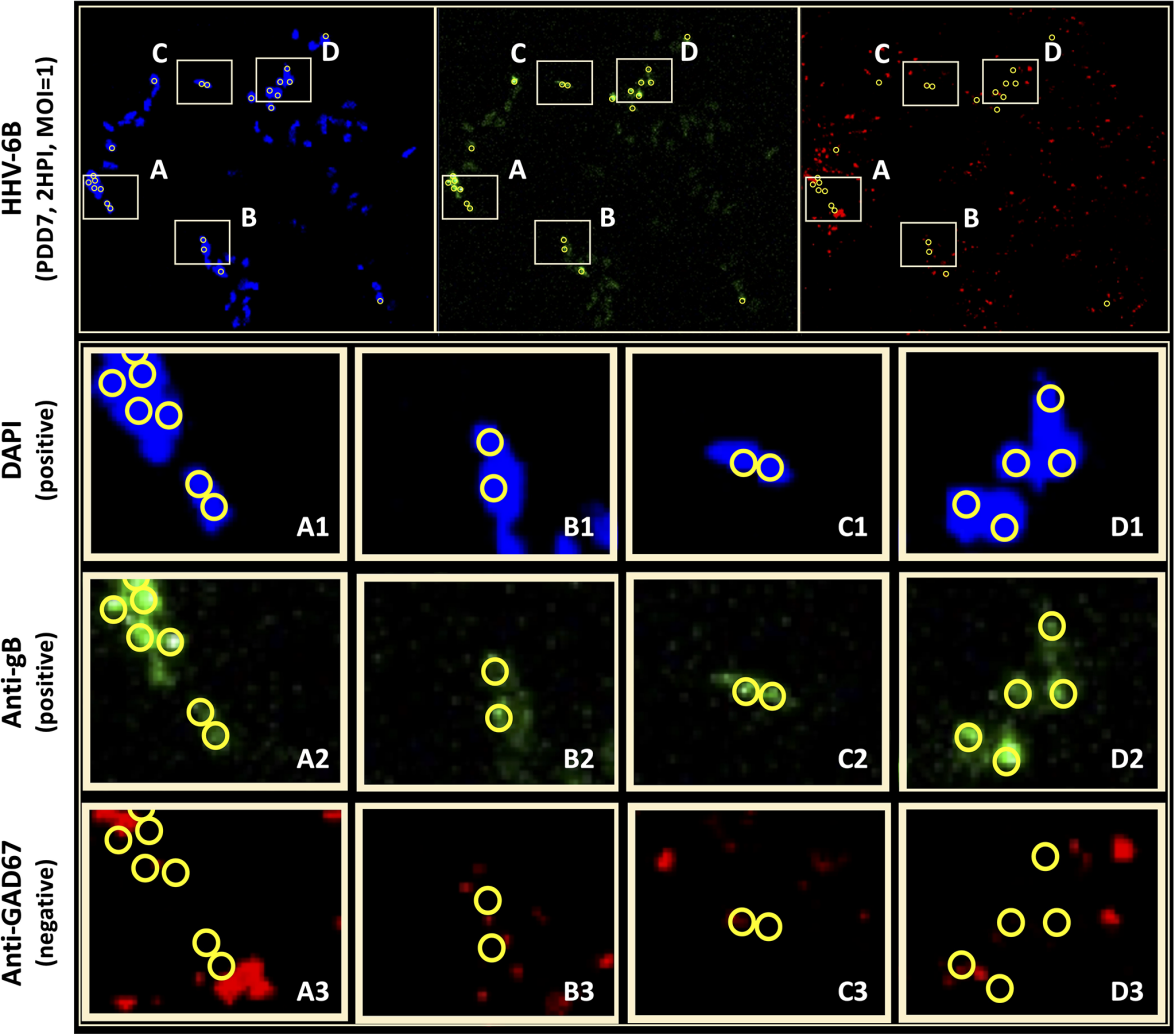
Anti-gB/DAPI fluorescence does not colocalize with GAD67-positive cells in mixed cultures. DAPI microscopy images of immunofluorescence and fluorescence staining of dHNSC at PDD7, MOI = 1, 2HPI challenged with HHV-6B. Out of multiple trials, only one anti-GAD67 positive culture displayed an anti-gB fluorescence signal indicating possible infection of GABA-containing neurons (*top*). However, upon closer examination of anti-gB cell clusters (regions A-D), it appears that DAPI positive cells (rows A1-D1) and anti-gB positive (rows A2-D2) cells are anti-GAD67 negative (row A3-D3) providing further evidence for GABAergic neuron resistance to HHV-6 infection. DAPI/anti-gB positive fluorescence is likely from adjacent glial cells or other neuronal neurotransmitter phenotypes in the mixed culture.

To determine whether neurons that synthesize gamma-aminobutyric acid (GABA) were susceptible to infection by HHV-6A or HHV-6B, a fluorescence antibody system against GAD67, a glutamate decarboxylase, which is responsible for the overwhelming majority (>90%) of GABA synthesis in the brain, was employed. (GABA is the major inhibitory neurotransmitter in the CNS). Cultures co-stained with DAPI and co-labeled with anti-GAD67 and the anti-gB fluoroprobes failed to show infection of GAD67-positive differentiated HNSCs by either HHV-6A (**Figure 8**, row A) or HHV-6B (**Figure 8**, row B). Given the unexpected results, multiple time-points were examined. However, anti-gB fluorescence was not detected at PDD7 or PDD14 (**Figure 8**) or under various MOIs (i.e., MOI=1 and 2). These experiments were repeated multiple times with the same results. For a single sample (out of two trials each done in triplicate, N=6, at PDD7 and, again, at PDD14) where differentiation of HNSC was driven toward GABA-producing cells, a few anti-gB fluorescence patches were observed in HHV-6B infected cultures (**Figure 9**).

Initial indications suggested that perhaps HHV-6B can indeed infect GAD67-positive cells. However, upon detailed analysis of immunofluorescence data, the colocalization of the DAPI and anti-gB signals (**Figure 9**: A1-D1 and A2-D2, respectively) do not coincide with anti-GAD67 signals, indicating that the anti-gB fluorescence emanates from nearby cells in these mixed cultures. Moreover, for many of the anti-GAD67 fluorescence there was no overlap with DAPI suggesting that these could be fluorescence signals from GABA-rich afferents (*see* **Figure 9**: A3, B3, C3, D3). Data indicate that >90% of cells were GAD67-positive in HHV-6A and HHV-6B infected cultures (N=6); yet, there was no evidence of coincidence between anti-GAD67 and anti-gB fluorescence.

### Immunofluorescence Indicates Both CD134 and CD46 Expression in GAD67-Positive Cells

**Figure 10:**
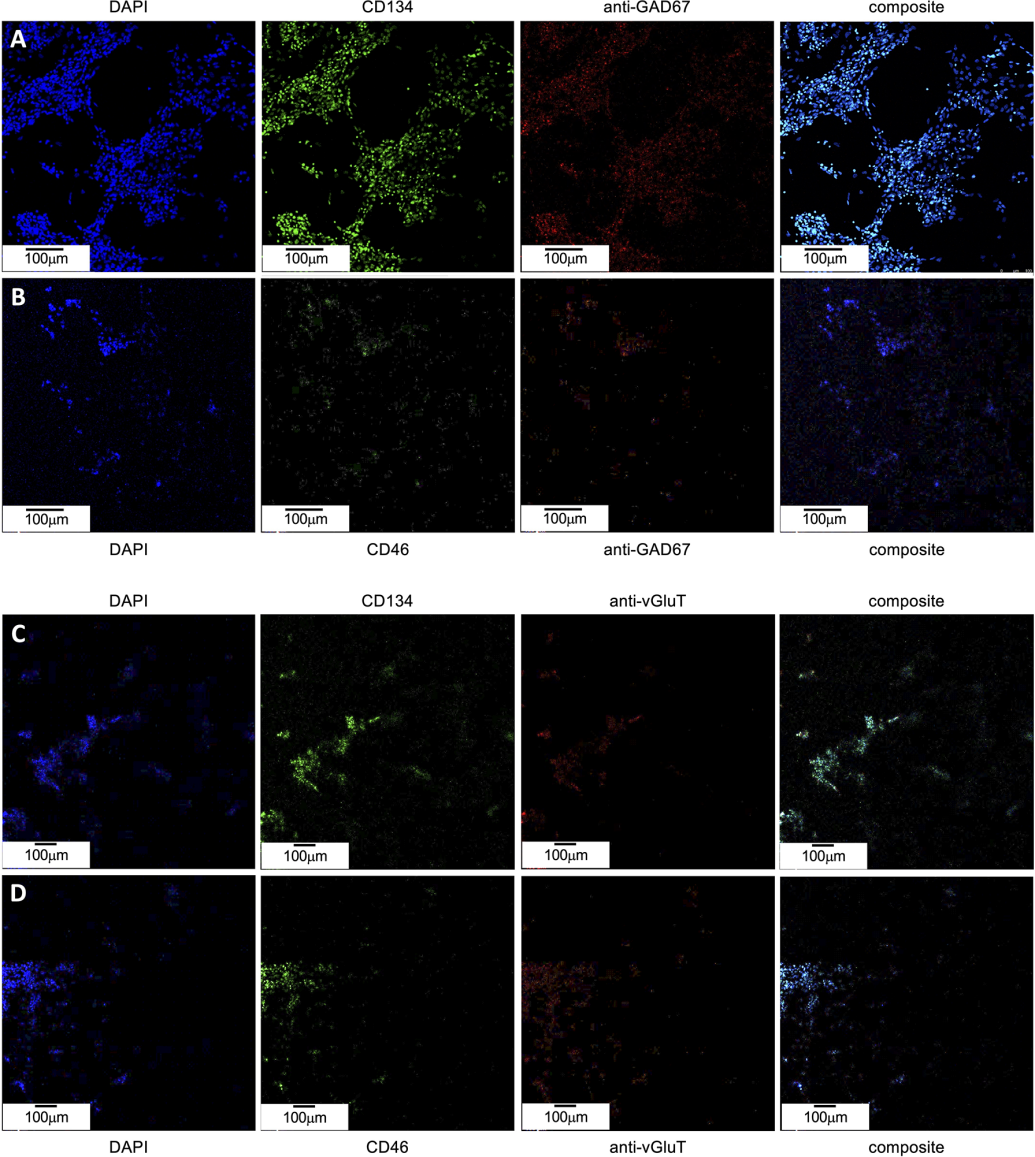
CD46 and CD134 colocalize with GAD67-positive and VGluT-positive differentiated H9 stem cells. **(A)** GAD67-positive (*red, mid-right*) dHNSCs at PDD7 stained with DAPI (*blue, left*) exhibit a coincident anti-CD134 immunofluorescence signal (*green, mid-left*). **(B)** GAD67-positive (*red, mid-right*) dHNSCs at PDD7 stained with DAPI (*blue, left*) also show colocalized anti-CD46 (*green, mid-left*). Thus, GAD-67-positive cells appear to express both CD134 and CD46 (*composites, right*). **(C)** VGluT-positive (*red, mid-right*) dHNSCs at PDD7 stained with DAPI (*blue, left*) also show colocalization of anti-CD134 immunofluorescence signal (*green, mid-left*). **(D)** VGluT-positive (*red, mid-right*) dHNSCs at PDD7 stained with (*blue, left*) show anti-CD46 immunofluorescence (*green, mid-left*). This indicates VGluT-positive cells also express CD134 and CD46 (*composites, right*). [Scale bar = 100 micron].

To determine if the apparent resistance of GAD67-positive cells to roseolovirus infection is due to low receptor expression, immunofluorescence was used to verify the coincidence of GAD67 with CD46 and CD134. CD46 is a receptor known to be used by HHV-6A for cell attachment and entry. CD134 is reported to be the preferred receptor for HHV-6B attachment and entry; however, it has also been shown that HHV-6B can use CD46. Failure to express these “cluster of differentiation” (CD) regulatory proteins in GAD67-positive cells would inhibit HHV-6A or HHV-6B infection. However, immunofluorescence data (**Figures 10A, B**) indicate that CD134 and CD46 are coincident with GAD67. These results are consistent with data from VGluT1-positive cultures (**Figures 10C, D**).

Immunofluorescence data indicate that receptor expression in GAD67-positive cells is greater than for VGluT1-positive cells (see **Figure 10**), in which productive roseolovirus infection is observed. Moreover, for all cultures examined (N=9), CD134 fluorescent markers appear in greater density than CD46.

**Figure 11:**
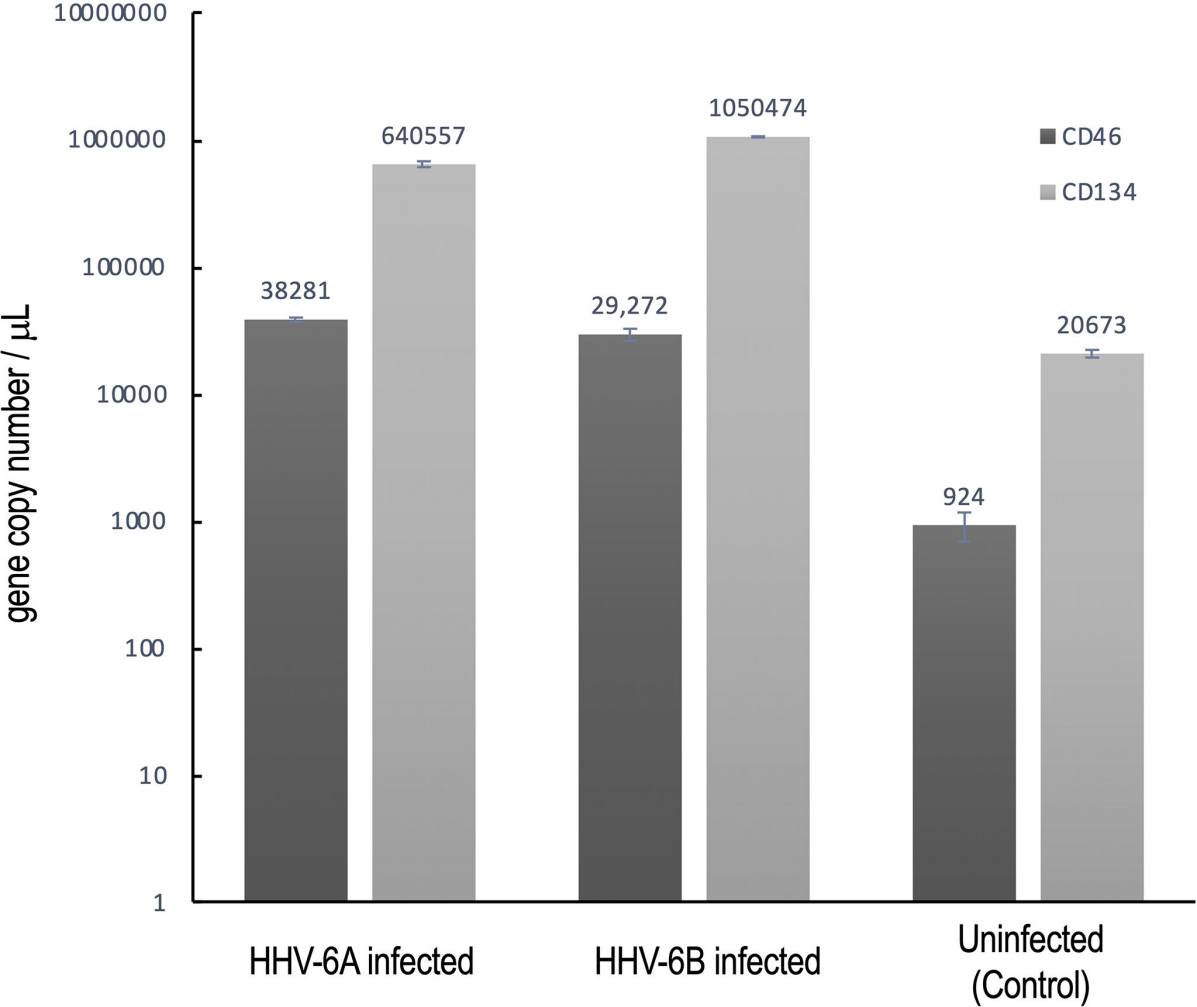
CD134 and CD46 gene expression in HHV6 infected cells. Using RT-qPCR CD134 and CD46 mRNA expression was determined for dHNSC (i.e., H9 cells) after infection with either HHV-6A or HHV-6B and compared to uninfected controls. After 2 hr post-infection, CD134 (light grey) and CD46 (dark grey) mRNA levels were elevated when compared to uninfected control cultures (far right); (N=6, p=0.0002). CD134 expression levels are greater than CD46 in HHV-6A and HHV-6B infected cultures; (N=6, p=0.0013). CD134 expression is also greater in uninfected cells. These data indicate that cells express CD134 at higher levels and that infection results in overexpression of both CD46 and CD134.

To confirm that CD134 is indeed expressed at higher levels than CD46, reverse transcription quantitative polymerase chain reaction (RT-qPCR) was used to determine relative transcription levels of CD134 versus CD46 in cultures infected with HHV-6A or HHV-6B and uninfected controls (**Figure 11**). These data indicate that CD134 expression is greater than CD46 expression in both HHV-6A and HHV-6B infected cultures as well as the uninfected control cultures (N=9, p=0.0001). However, neither CD134 nor CD46 expression are significantly different between HHV-6A versus HHV-6B infected cultures. Interestingly, expression of both receptors increases significantly during roseolovirus infection (when compared to uninfected controls). During HHV-6A infection, a significant increase in both CD134 and CD46 expression is observed over uninfected controls (N=6, p=0.0002). Likewise, HHV-6B infections results in a significant increase in CD134 and CD46 expression over uninfected controls (N=6, p=0.0013). Results held in mixed cultures and are independent of whether cultures are dominated by glutamatergic, dopaminergic, or GABAergic cell types.

### Immunofluorescence and qPCR Show That HHV-6A Induces a TLR9 and IL-10 Response

**Figure 12:**
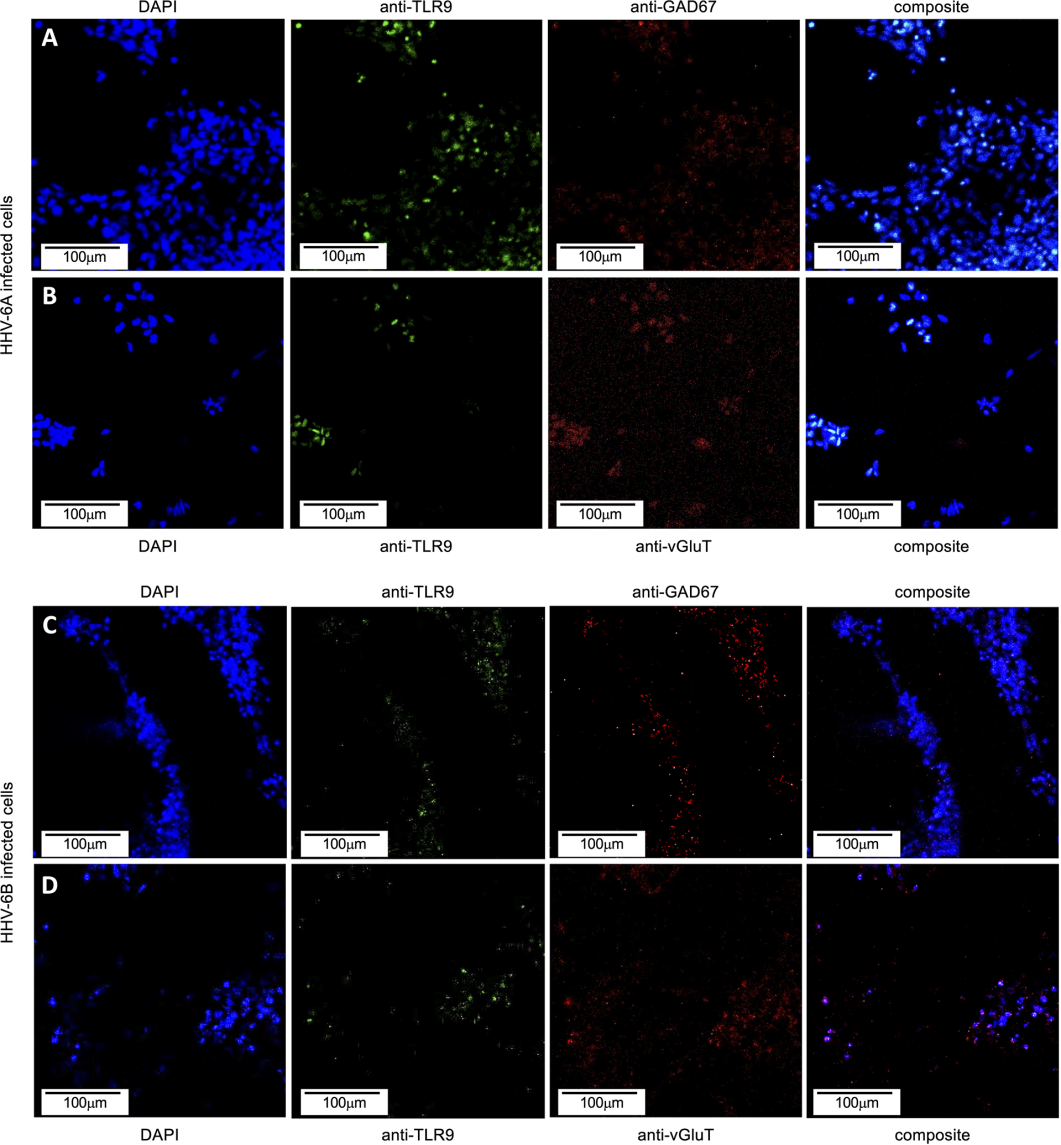
TLR9 in HHV6 infected excitatory (VGluT-positive) and inhibitory (GAD67-positive) cells. Immunofluorescence assays indicate that both HHV-6A and HHV-6B infected dHNSCs express TLR9. **(A)** DAPI stained cells (*blue, left*) emitting GAD67-positive immunofluorescence signals (*red, mid-right*) also show signal for anti-TLR9 fluoroprobes (*green, mid-left*) during HHV-6A infection. **(B)** HHV-6A infected VGluT-positive neurons (*red, mid-right*) stained with DAPI (*blue, left*) also show coincident TLR9 immunofluorescence (*green, mid-right*). **(C)** DAPI stained cells (*blue, left*) emitting GAD67-positive immunofluorescence signals (*red, mid-right*) also show signal for anti-TLR9 fluoroprobes (*green, mid-left*) during HHV-6B infection. **(D)** HHV-6B infected VGluT-positive cells (*red, mid-right*) stained with DAPI (*blue, left*) also show coincident TLR9 fluorescence (*green, mid-right*). [Infections in A-D were performed seven days post-differentiation day (PDD7). [Scale bar = 100 micron].

Toll-like receptors (TLRs) act as early sensors for pathogen detection and initiation of biochemical cascades associated with cellular immunological response to microbial infection (23). Although TLR9 is a known pro-inflammatory receptor in immune cells, in non-immune cells, including neurons, TLR9 may play a role in energy metabolism to protect cells during infection (22). To determine if TLR9 expression is impacted during HHV-6A or HHV-6B infection in dHNSC, immunofluorescence was used (*see* **Figure 12**). An anti-TLR9 antibody system was employed in conjunction with anti-VGluT1 and anti-GAD67 during separate infections with HHV-6A and HHV-6B. When compared to uninfected controls, TLR9 signals showed no significant difference in fluorescence emissions over uninfected controls in HHV-6B infected cells. However, there does appear to be a higher density of TLR9 signal in HHV-6A infected cell cultures than in cultures infected with HHV-6B (**Figure 12**).

**Figure 13:**
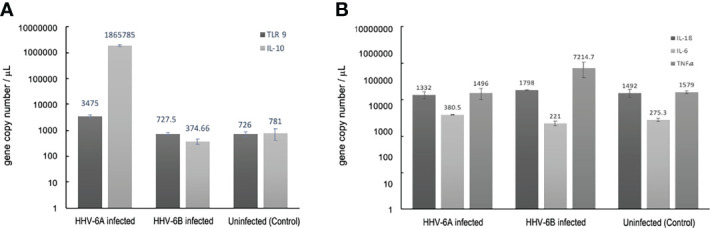
Cellular cytokine responses to HHV6 infection in dHNSCs *via* RT-qPCR. **(A)** TLR9 gene expression levels (i.e., mRNA) are elevate in HHV-6A infected cultures;(N=3, p=0.0150) while no significant increase in TLR9 gene expression in HHV-6B infected dHNSCs when compared to uninfected controls (*dark grey*). Likewise, a significant increase in IL-10 is observed in HHV-6A infected cultures;(N=3, p=0.0029), while no significant increase in IL-10 gene expression is observed in HHV-6B infected dHNSCs (*light grey*). **(B)** Gene expression levels of IL-6 are slightly elevated in HHV-6 infected cultures (*light grey*), while no significant difference in IL-6 expression in HHV-6B infected dHNSCs is observed as compared to uninfected controls. TNFα expression is elevated in HHV-6B infected dHNSCs. No significant difference is observed in HHV-6A infected cells when compared to uninfected controls (*medium grey*). No change in IL-1β is noted for either HHV-6A or HHV-6B infected cells (*dark grey*).

To quantitatively examine TLR9 expression in HHV-6A infected versus HHV-6B infected dHNSC, RT-qPCR was employed (**Figure 13A**, dark grey). Consistent with observations from the immunofluorescence assays, which target protein, mRNA levels for TLR9 in HHV-6B infected cell cultures were not significantly different from uninfected control cultures. However, TLR9 gene expression was significantly upregulated in HHV-6A infected cell cultures over HHV-6B infected cell cultures (N=3, p=0.02986) as well as uninfected controls (N=3, p=0.0001). These RT-qPCR data (**Figure 13A**) are consistent with results from immunofluorescence (**Figure 12**).

Since early pro-inflammatory responses initiated by TLR9 (e.g., regulation of IL-1β and TNFα) may be suppressed by IL-10, RT-qPCR was used to determine IL-10 transcript levels under uninfected control conditions and during HHV-6A versus HHV-6B infections (**Figure 13A**, light grey). IL-10 mRNA expression levels were not significantly different between the uninfected controls and HHV-6B infected cultures. However, coincident with higher TLR9 expression levels, IL-10 mRNA levels were significantly higher in HHV-6A infected cells than in HHV-6B infected cells (N=3, p=0.0003) and uninfected controls (N=3, p=0.0001).

There are multiple steps in the immunological response between activation of TLR9 and IL-10 suppression of inflammatory cytokines. RT-qPCR was also used to explore the regulation of other pathway intermediates in HHV-6A versus HHV-6B infections compared to uninfected controls.

### Regulation of Other Immunological Factors During HHV-6 Infection as Detected by qPCR

Given a significant upregulation in the anti-inflammatory cytokine, IL-10, during HHV-6A infection, it was prudent to examine the expression of pro-inflammatory cytokine levels (e.g., IL1β, TNFα). Again, RT-qPCR was performed to determine the impact of HHV-6A versus HHV-6B infection on the expression pro-inflammatory cytokines (**Figure 13B**). When compared to uninfected controls, there was no appreciable difference in IL-1β expression levels between HHV-6A or HHV-6B infected cultures (**Figure 13B**, dark grey). HHV-6B infected cells exhibited a marked increase in TNFα expression while HHV-6A infected cells showed no significant difference in TNFα over control (**Figure 13B**, medium grey). Neither HHV-6A nor HHV-6B infected cell cultures exhibited notable increases in IL-6 expression (**Figure 13B**, light grey).

### Regulation of Cellular Growth Factors Detected by qPCR During HHV-6 Infection

**Figure 14:**
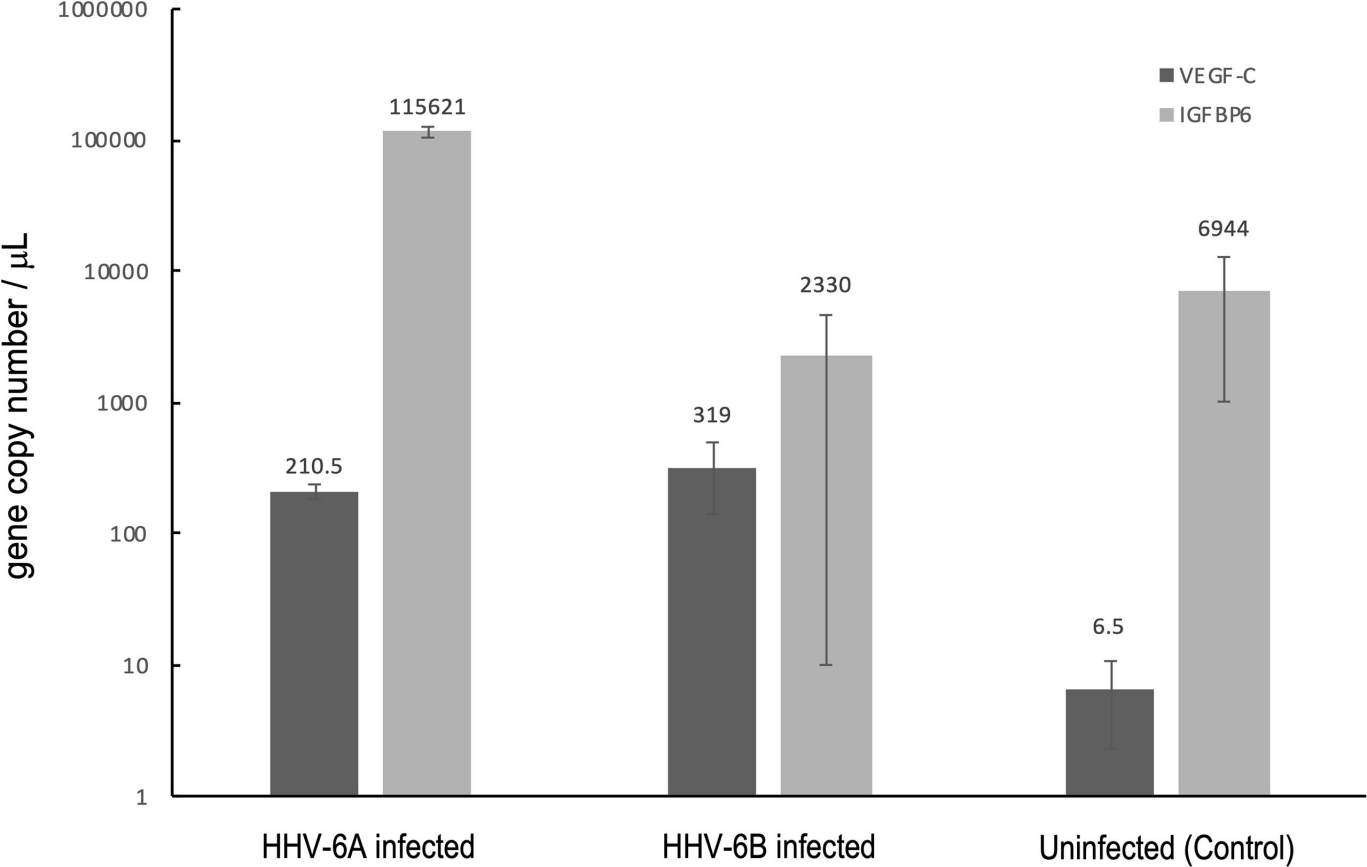
Growth factor responses to HHV6 infection in dHNSCs *via* RT-qPCR. Expression levels of vascular endothelial growth factor C (VEGF-C) and insulin-like growth factor binding protein 6 (IGFBP6) were measured *via* RT-qPCR during infection with HHV-6A and HHV-6B. Both HHV-6A(N=3, p=0.0009) and HHV-6B (N=3, p=0.0022) infected dHNSCs show elevated expression of VEGF-C compared to uninfected controls (*dark grey*); (N=3, p=0.0000). HHV-6A infection of dHNSCs also results in increased expression of IGFBP6; (N=3, p=0.0064) while HHV-6B infection does not (*light grey*).

In addition to evoking cytokine responses, viruses are known to upregulate the expression of select cellular growth factors, including insulin-like growth factor binding protein 6 (IGFBP6), which has been shown to be upregulated during HHV-6A infection (24), and vascular endothelial growth factor-C (VEGF-C), which was shown to be upregulated in HHV-1 (a.k.a., HSV-1) infection (25). To determine if these two growth factors are upregulated in response to HHV-6A or HHV-6B infection in dHNSc, RT-qPCR was used to measure transcript levels in infected versus uninfected controls (**Figure 14**). For IGFBP6, results from HHV-6B infected cells compared to control were inconclusive; however, IGFBP6 expression was significantly upregulated (N=3, p=0.0064) in HHV-6A infected cultures (**Figure 14**, light grey). Both HHV-6A (N=3, p=0.0009) and HHV-6B (N=3, p=0.0022) infected cell cultures exhibited increased VEGF-C levels over uninfected control. However, there was no significant difference between VEGF-C levels in HHV-6A versus HHV-6B infected cultures (**Figure 14**, dark grey).

## Discussion

Due to initial perceptions that HHV6 isolates group into one virus species (26) and the later revelation that there are two distinct viruses, the earliest literature (prior to 2012) does not specify cell tropism differences between HHV-6A versus HHV-6B (27). To adequately evaluate proposed models for HHV6-induced seizure induction, it is necessary to unravel the details regarding HHV-6A versus HHV-6B cell tropism and the cellular responses (e.g., immunological) in nerve cells infected with HHV-6A versus HHV-6B (28). Our results and prior work regarding HHV-6A and HHV-6B cell tropism indicate that HHV6 infects nerve cells (11, 27, 29, 30).

### HHV-6A and HHV-6B Infect Both Neurons and Glia With Different Levels of Cytopathology

Specifically, results from immunofluorescence data show that HHV-6A or HHV-6B can infect cells cultured from H9 human embryonic stem cell-derived neural stem cells (see **Figures 1** and **2**). Results from qPCR and TEM clearly indicate that productive infection of dHNSC occurs (**Figure 3**) when either HHV-6A or HHV-6B is perfused into dHNSC cultures at a MOI of 0.5, 1.0, or 2.0. Light microscopy and viable cell counts indicate differential impacts of HHV-6A versus HHV-6B on cell cultures (e.g., PDD7, 2HPI, MOI=1). For example, it appears HHV-6A induces more severe cytopathic effects than HHV-6B for the same day post-differentiation and post-infection end-point (**Figure 4**). For MOIs examined, both HHV-6A and HHV-6B induce cell clumping and detachment from surface substrate; however, the degree of cell aggregation and detachment is greater in HHV-6A infected cultures at both PDD7 and PDD14 (2HPI and 24HPI). Indeed, cells with intact processes are still visible in HHV-6B infected cultures even at MOI=2 at PDD14 (see **Figure 4**). Moreover, syncytia formation was only observed during HHV-6A infections of dHNSC (**Figure 5**). These results suggest that HHV-6A infection may be more detrimental to cell culture than infection with HHV-6B. This is consistent with prior work suggesting that HHV-6A is more virulent in glia (31). Although models for HHV-6 induced MS highlight HHV-6A infection in myelin-producing oligodendrocytes as a putative etiologic mechanism (32), some models for HHV-6 induced epilepsy specify virus infection of neurons. Indeed, current views link HHV-6A infection to MS, while HHV-6B infection is associated with predisposition for epilepsy (33; 34; 35).

### HHV-6A and HHV-6B Infect vGluT^+^ and DA^+^ Cells but Neither Virus Infects GAD67^+^ Cells

Although an early study demonstrated that HHV-6 infects glial cells (16), only recently was it shown that both HHV-6A and HHV-6B can infect neurons (17). This latter study only showed HHV-6A and HHV-6B infection in Purkinje cells, which are unique.

Results from our study provide additional details regarding cell tropism for specific neuronal neurotransmitter phenotypes. Immunofluorescence microscopy shows that VGluT1-positive cells (i.e., glutamatergic neurons) and DA-positive cells (i.e., dopaminergic neurons) are susceptible to either HHV-6A or HHV-6B (**Figures 6** and **7**). Although both glutamatergic and dopaminergic cells are susceptible to both viruses, infection assays failed to demonstrate that GAD67-positive cells (i.e., GABAergic neurons) were susceptible to either virus. We were initially skeptical of these results since the aforementioned study showed susceptibility of Purkinje cells, which are GABAergic, to both HHV-6A an HHV-6B (17). However, repeated trials using immunofluorescence suggest that GAD67-positive cells derived from dHNSC are resistant to infection by these viruses (**Figure 8**). In one infection trial with HHV-6B, a sparse anti-gB signal was observed in mixed cultures containing GAD67-positive cells (**Figure 9**, top row, middle panel). However, with higher magnification and a more detailed image analysis, anti-GAD67 emissions do not overlap with anti-gB fluorescence. This suggests that HHV-6B is likely infecting cells adjacent to GABA neurons in mixed cultures (e.g., glial cells) or that anti-gB signals are emanating from afferents from other infected cell types onto GABAergic cells (e.g., axonal-somatic inputs).

One possibility for the apparent inability of HHV-6A or HHV-6B to infect GABAergic cells is that these GAD67-positive dHNSC do not express CD134 or CD46, the cell surface receptors responsible for HHV6 attachment and entry into host cells (DeBolle et al., 2005; 10). Upon testing this hypothesis using both immunofluorescence (**Figure 10**) and RT-qPCR (**Figure 11**), results showed that GAD67-positive cells are not only expressing CD134 and CD46, but CD134 expression was higher under all conditions (i.e., uninfected, HHV-6A infected, HHV-6B infected). Although the relative binding affinity of HHV-6A versus HHV-6B on each of these cell surface receptors is somewhat unclear (36; 37), it is known that HHV-6B can use CD46 but preferentially binds to CD134. Thus, it is reasonable to suggest that HHV-6B has an advantage over HHV-6A in terms of receptor availability, attachment, and entry into susceptible hosts. Despite this potential advantage in receptor availability for HHV-6B, our results suggest that HHV-6A infection results in more severe CPEs at earlier stages of infection of cell cultures (see **Figures 4** and **5**). Again, this would indicate that HHV-6A is more virulent virion-for-virion when compared with HHV-6B. These results are supported by prior work suggesting that infection of oligodendrocytes with HHV-6A induces cell lysis while HHV-6B does not (33). This also supports the idea that HHV-6A may be involved in direct demyelination of axons *via* lysis of oligodendrocytes leading to MS, while neuronal dysfunction-based hypotheses of HHV-6 induced epilepsy may be more complex.

### Cytokine Responses Differ Between Cell Cultures Infected With HHV-6A Versus HHV-6B

Viral infections induce inflammatory responses in both immune cells and non-immune cells. Determining the extent to which HHV-6A versus HHV-6B infections upregulate the expression of pro-inflammatory factors (e.g., cytokines) could provide additional insights into relative virulence. To quantify the immunological impacts of HHV-6A versus HHV-6B infection in nerve cell cultures, experiments were performed to investigate potential changes in cytokine expression levels during HHV-6 infections versus uninfected controls. In addition to CD46 (or CD134) expression for virus entry, toll-like receptor 9 (TLR9) may be activated by roseolovirus infection (38). TLR9 is a major pattern recognition receptor for bacteria and DNA viruses (39). Prior work showed that HHV-6A infection in CD4^+^ T cells not only activates TLR9 but also increases its expression (40). In cultures of dHNSC, our results show a more robust TLR9 fluorescence signal after HHV-6A infection compared to HHV-6B infected cultures (**Figure 12**).

This is supported by RT-qPCR as gene copie numbers are higher in HHV-6A infections (**Figure 13A**).

This is more evidence of differential impact of HHV-6A versus HHV-6B on susceptible nerve cells.

These results are consistent with prior work that show an increase in TLR9 expression in microglia and astrocytes from both mice and humans upon infection with HHV-6A (38). TLR9 activation is known to coincide with the activation (and upregulated expression) of cytokines including: interleukin-1β (IL-1β), tumor necrosis factor α (TNFα), and IL-6 (41). In response to activation of pro-inflammatory factors (e.g., TLR9, IL-1β, TNFα), IL-10 may be upregulated as an anti-inflammatory reaction, especially in neurons (22). Interestingly, our results show a remarkable increase in IL-10 expression concomitant with the upregulation of TLR9 gene expression in cultures infected with HHV-6A. IL-10 upregulation may serve to inhibit a robust IL-1β pro-inflammatory response in HHV-6A infected cells (see **Figure 13A**). This IL-10 increase may account for negligible change in IL-1β after HHV-6A infection (**Figure 13B**). Upon examination of pro-inflammatory cytokine expression (i.e., IL1β and TNFα), IL-1β was not elevated under any condition; however, HHV-6B infection appears to increase TNFα (**Figure 13B**). It would be anticipated that in addition to attenuating IL-1β, IL-10 would also temper TNFα levels (42). However, our results do not support this in dHNSC infected with HHV-6B.

More detailed time-course of infection studies will be needed to relate IL-10 activation/expression with changes in other cytokine levels (e.g., IL-1β and TNFα). Interestingly, IL-6 may act as either a pro-inflammatory cytokine (like IL1β and TNFα) under conditions of chronic inflammation or as an anti-inflammatory cytokine (like IL-10) during acute inflammatory responses (43). Results from our *in vitro* HHV6 infection assays yield mixed results with regards to IL-6 (**Figure 13**). Specifically, IL-6 expression is greater in HHV-6A infection but shows no significant change in HHV-6B infected cells. Although it has been reported that general neuroinflammatory responses can lead to encephalitis and herpesvirus-induced seizures, suppression of neuroinflammatory pathways during HHV-6 infection has led to other hypotheses regarding mechanisms of HHV6-induced epileptogenesis (44).

### Growth Factor Levels Differ Between Cell Cultures Infected With HHV-6A Versus HHV-6B

In addition to cytokine responses, it is known that select cellular growth factors can be activated or upregulated during viral infection (45, 46). Both vascular endothelial growth factor-C (VEGF-C) and insulin-like growth factor binding protein-6 (IGFBP-6) have been shown to be upregulated during herpesvirus infections (24). VEGFs are responsible for activating endothelial cells by attaching to vascular endothelial growth factor receptors on the cell surface (47). VEGF-C appears to play a role in the pathogenesis of several viral diseases, including those involving HHV nervous system infections.

For example, it was shown that HHV-1 (a.k.a., HSV1) infection can enhance VEGF-C expression (25). It was also shown that patients with virus-positive encephalitis exhibit higher levels of VEGF-C in serum than patients with virus-negative encephalitis (48).

Specific to roseoloviruses, it has been reported that VEGF-C is continuously activated in HHV-6 infected astrocytes at twice the level compared to uninfected controls (24). Our results show that both HHV-6A and HHV-6B infection can elevate VEGF-C levels in dHNSC. This is notable since VEGF-C protein levels were found to be elevated in the temporal neocortex of patients with temporal lobe epilepsy (49). It is notable for our results that both HHV-6A and HHV-6B elevate VEGF-C mRNA to comparable levels over uninfected controls (**Figure 14**, *dark grey*). Likewise, it has been shown that IGFBPs are overexpressed in response to viral infection as a result of inflammatory cytokine activation (50). For example, HHV-6A has been shown to increase IGFBP-6 expression in astrocytes (24). Our results indicate that IGFBP-6 is also overexpressed in HHV-6A infected dHNSC but not in cultures infected with HHV-6B (**Figure 14**, *light grey*).

### Implications of Results for Current Models of HHV-6 Induced Epileptogenesis.

Several models are proposed as potential mechanisms by which HHV-6 induced seizures can lead to epilepsy (i.e., epileptogenesis). Although neural encephalitis may emerge from primary HHV-6 infections leading to seizure (51–53), sub-inflammatory mechanisms have also been proposed. For example, there is some *in vitro* evidence to suggest that HHV-6 infection of astrocytes can lead to dysfunction in glutamate reuptake from the synapse, leading to hyperexcitation of glutamatergic pathways and thus seizure (14, 54). Beyond this *glutamate reuptake dysfunction hypothesis*, other studies have suggested that reactivation of HHV-6 from latency may result in lysis of glutamatergic cells resulting in excess release of glutamate into neuronal circuits associated with seizure induction (e.g., mesial temporal lobe neural networks). This has been described as an *excitotoxicity model* (55–58). If results observed in cultured dHNSC are generalizable to *in vivo* conditions, then a glutamatergic excitotoxicity model would be supported by gross glutamatergic cell loss during HHV-6A infection.

Another hypothesis is that GABAergic interneurons which modulate glutamatergic pathways could be selectively targeted by HHV-6A or HHV-6B. Disruption of the inhibitory modulation of glutamatergic neurons would, in turn, lead to excess glutamate release and subsequent seizure.

If our results regarding a lack of susceptibility of GABAergic neurons to HHV6 are generalizable to *in vivo* conditions, then this *inhibitory (inter)neuron dysfunction hypothesis* could be ruled out. Alternatively, HHV6-induced epileptogenesis may involve more than one of these proposed models or another yet to be described mechanism. For example, cholinergic pathways from the peduncular pontine nuclei (PPN) or other tracts may also contribute to seizure induction by hyperexciting glutamatergic targets or inhibiting modulating interneurons. Finally, it is also necessary to explore impacts of roseolovirus infection on neuronal and neural network signaling. This is the subject of ongoing research in our lab.

## Conclusions

Understanding differences in the susceptibility as well as the immunological responses of distinct neuronal neurotransmitter phenotypes responses to HHV-6A versus HHV-6B infection will permit a more critical evaluation of models that seek to explain HHV6-induced neurological disorders (59). Combining results presented here from cytokine/growth factor regulation and physiological studies, there is ample evidence to conclude that there are differential impacts of HHV-6A versus HHV-6B infection on nerve cell viability, structure, and function in cultured cells. How this relates to neuronal signaling and neural circuit behavior other *in vitro* and *in vivo* models is the subject of ongoing work in the lab. Along with prior reports that support our results, we suggest that each virus may exhibit different levels of virulence on select cell types with HHV-6A being more virulent despite the apparent advantage of HHV-6B to more readily infect cells with high densities of CD46 and CD134 expression.

## Data Availability Statement

The original contributions presented in the study are included in the article/**Supplementary Materials**. Further inquiries can be directed to the corresponding author.

## Author Contributions

RC is the principal investigator responsible for the overall work on this project. RC was the principal author of the manuscript and was involved in all aspects of data analysis as well as overseeing the work of student and postdoctoral co-authors. EB did all cell culturing and immunofluorescence assays as well as qPCR. MF assisted in cell culture and data analysis. JW conducted image analyses in support of this work. All authors contributed to the article and approved the submitted version.

## Funding

The authors thank the Arkansas Bioscience Institute for a 1-year equipment andsupply grant to support this research and the U.S. National Science Foundation for supporting the work of JW via an NSF Research Experiences for Undergraduates (REU) grant (award no.1659858; PI-Ceballos). The work was supported in part by an NSF DBI Biology Integration Institute (BII) grant (award no. 2119968; PI-Ceballos).

## Conflict of Interest

The authors declare that the research was conducted in the absence of any commercial or financial relationships that could be construed as a potential conflict of interest.

## Publisher’s Note

All claims expressed in this article are solely those of the authors and do not necessarily represent those of their affiliated organizations, or those of the publisher, the editors and the reviewers. Any product that may be evaluated in this article, or claim that may be made by its manufacturer, is not guaranteed or endorsed by the publisher.
