# Supplementary figures and images for: Differential Impacts of HHV-6A versus HHV-6B Infection in Differentiated Human Neural Stem Cells
Source: Front Immunol. 2022 Jul 13;13:847106. doi: 10.3389/fimmu.2022.847106 (PMC9326508; doi:10.3389/fimmu.2022.847106)

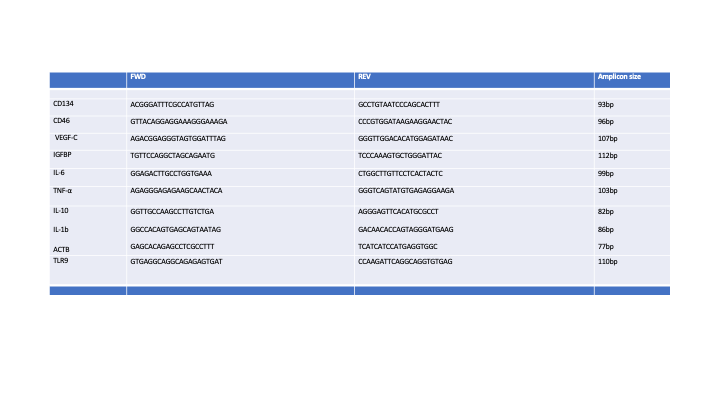

Supplement: Supplementary file 1 [file Image_1.tiff]
